# Naringenin as potent anticancer phytocompound in breast carcinoma: from mechanistic approach to nanoformulations based therapeutics

**DOI:** 10.3389/fphar.2024.1406619

**Published:** 2024-06-18

**Authors:** Deena Elsori, Pratibha Pandey, Seema Ramniwas, Rahul Kumar, Sorabh Lakhanpal, Safia Obaidur Rab, Samra Siddiqui, Ajay Singh, Mohd Saeed, Fahad Khan

**Affiliations:** ^1^ Faculty of Resilience, Rabdan Academy, Abu Dhabi, United Arab Emirates; ^2^ Centre of Research Impact and Outcome, Chitkara University, Rajpura, India; ^3^ University Centre of Research and Development, University Institute of Biotechnology, Chandigarh University Gharuan, Mohali, India; ^4^ Chitkara Centre for Research and Development, Chitkara University, Himachal Pradesh, India; ^5^ School of Pharmaceutical Sciences, Lovely Professional University, Phagwara, India; ^6^ Department of Clinical Laboratory Sciences, College of Applied Medical Science, King Khalid University, Abha, Saudi Arabia; ^7^ Department of Health Service Management, College of Public Health and Health Informatics, University of Ha’il, Ha’il, Saudi Arabia; ^8^ School of Applied and Life Sciences, Uttaranchal University, Dehradun, India; ^9^ Department of Biology, College of Science, University of Hail, Ha’il, Saudi Arabia; ^10^ Center for Global Health Research Saveetha Medical College, Saveetha Institute of Medical and Technical Sciences, Chennai, India

**Keywords:** cancer therapeutics, naringenin, phytochemicals, breast cancer, nanoformulations

## Abstract

The bioactive compounds present in citrus fruits are gaining broader acceptance in oncology. Numerous studies have deciphered naringenin’s antioxidant and anticancer potential in human and animal studies. Naringenin (NGE) potentially suppresses cancer progression, thereby improving the health of cancer patients. The pleiotropic anticancer properties of naringenin include inhibition of the synthesis of growth factors and cytokines, inhibition of the cell cycle, and modification of several cellular signaling pathways. As an herbal remedy, naringenin has significant pharmacological properties, such as anti-inflammatory, antioxidant, neuroprotective, hepatoprotective, and anti-cancer activities. The inactivation of carcinogens following treatment with pure naringenin, naringenin-loaded nanoparticles, and naringenin combined with anti-cancer agents was demonstrated by data *in vitro* and *in vivo* studies. These studies included colon cancer, lung neoplasms, breast cancer, leukemia and lymphoma, pancreatic cancer, prostate tumors, oral squamous cell carcinoma, liver cancer, brain tumors, skin cancer, cervical and ovarian cancers, bladder neoplasms, gastric cancer, and osteosarcoma. The effects of naringenin on processes related to inflammation, apoptosis, proliferation, angiogenesis, metastasis, and invasion in breast cancer are covered in this narrative review, along with its potential to develop novel and secure anticancer medications.

## 1 Introduction

Cancer is a serious and frequently fatal disease and one of the leading causes of death globally. It is a complex pathological condition that can be induced by oxidative stress, genetic mutations, uncontrolled cell proliferation due to defects in the cell cycle or apoptosis, exposure to harmful radiation, and pollution. Unfortunately, established anticancer treatments, such as chemotherapy and radiation therapy, pose severe side effects in patients with cancer. Lifestyle is one of the many factors affecting cancer incidence (Bakar et al., 2014; Martincorena and Campbell, 2015; Bhat et al., 2017; Hayes et al., 2020). Research indicates that diets high in plant-based and bioactive substances can reduce the incidence and spread of cancer. Novel medications and treatment technologies are currently being developed through creative research on cancer treatments (Roy et al., 2022). Bioactive compounds originating from plants have been the subject of an increasing number of recent studies aimed at discovering new therapeutic agents from natural products (Connor and Lee, 2010).

Increased fruit and berry intake, particularly citrus fruit intake, may help prevent cancer and slow its spread (Wallace et al., 2020; Akbari et al., 2022). Fruits contain polyphenolic flavonoid components, which are the primary active ingredients (Lima et al., 2014). Flavonoids have been shown to exhibit potent anticancer properties through their role as antioxidants; modulation of ROS-scavenging enzyme activity; upregulation of cell cycle arrest, autophagy, and apoptosis; and downregulation of inflammation, proliferation processes, and metastasis formation (Wei et al., 2018; Majidinia et al., 2019; Hazafa et al., 2020; Sun et al., 2023). Naturally occurring citrus flavonoid naringenin (NGE) possesses a wide range of pharmacological properties, including anti-inflammatory, antioxidant, anti-ulcer, anti-apoptotic, and anti-carcinogenic effects (Joshi et al., 2018; Wojnar et al., 2018; Bakar et al., 2019; Ucar and Goktas, 2023). Additionally, naringenin has been reported to increase cell apoptosis and growth arrest of tumor cells, including those that cause cervical, bladder, prostate, and breast cancers (Shi et al., 2021). To our best, very limited reviews have summarized the anticancer potential of naringenin against breast cancer, therefore, our research has been directed towards understanding the processes by which naringenin, either alone or in conjunction with other therapeutic drugs, influences the advancement of cancer. Additionally, we presented various nanoformulations that are employed for the regulated distribution of naringenin in malignancies.

We have included a literature review (approximately more than 500 manuscripts) of data from 2010 to 2024 from various sources including Google Scholar (https://scholar.google.com/), PubMed (https://pubmed.ncbi.nlm.nih.gov/), and NCBI (https://www.ncbi.nlm.nih.gov/). We have also used *in silico* pathway databases and software, including PUBCHEM (https://pubchem.ncbi.nlm.nih.gov/) and SWISS-ADME (http://www.swissadme.ch/), to analyze the pharmacokinetic potential of naringenin as well as its crucial targets against several carcinomas.

## 2 Structure of naringenin

Power and Tutin (1907) discovered naringenin (2, 3-dihydro-5,7-dihydroxy-2-(4-hydroxyphenyl)-4H-1-benzopyran-4-one) as a flavanone (Zaidun et al., 2018). Dimethyl sulfoxide and ethanol are two chemical solvents that can dissolve naringenin, a hydrophobic molecule having a molecular weight of 272.25 g/mol (C_15_H_12_O_5_). It is a byproduct of the hydrolysis of naringenin or narirutin and is mostly found as an aglycone; however, it can also occur in glycosylated and neohesperidoside forms (Table 1). Oranges, tomatoes, lemons, and grapefruits are the primary producers of naringenin (Kiran et al., 2017). Naringenin is primarily transformed into its aglycone form, naringin, in the human body because of its limited absorption through the gastrointestinal system. Flavonoids are a 15-carbon skeleton, having two benzene rings connected by a three-carbon linking chain. Naringenin was more effective than naringin because of the steric barrier the two rhamnose units provided. The bitter flavor of grapefruit juice is attributed to NGE 7-O-neohesperidoside (Stabrauskiene et al., 2022).

**TABLE 1:** Physicochemical descriptors and pharmacokinetic properties of naringenin (Source: PubChem, https://pubchem.ncbi.nlm.nih.gov/compound/439246).

Physiochemical properties	
Structure	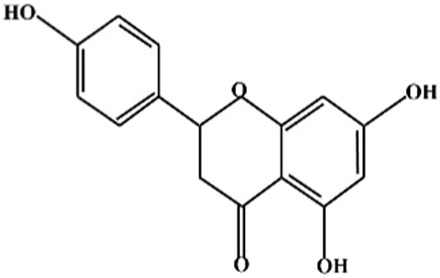
Compound CID	439,246
Chemical Formulae	C_15_H_12_O_5_
Molecular weight	272.25 g/mol
Number of H bond Acceptor	5
MLOGP	0.71
Number of H bond donor	3
Pharmacokinetics	
GI absorption	High
BBB Permeant	No
P-gp substrate	Yes
CYP1A2 inhibitor	Yes
CYP2C19 inhibitor	No
CYP2C9 inhibitor	No
CYP2D6 inhibitor	No
CYP3A4 inhibitor	Yes
Log Kp (skin permeation)	−6.17 cm/s

Lipinski rule: Molecular weight ≤500; Number of Hydrogen bond donor ≥5; Number of Hydrogen bond acceptor≥10; mLogP≤5.

CYP1A2: Member of the cytochrome P450 superfamily of enzymes. The cytochrome P450 proteins are monooxygenases that catalyze many reactions in drug metabolism and synthesize cholesterol, steroids, and other lipids.

CYP2C19: Principal enzyme involved in the hepatic metabolism of drugs.

CYP2C9: Enzyme breaks down (metabolizes) compounds including steroid hormones and fatty acids.

CYP2D6: Polymorphic drug-metabolizing enzyme.

CYP3A4: drug metabolizing enzymes mainly in liver and intestine.

Log Kp: Ability of compounds to penetrate the skin and induce toxicity. Negative Log kp implies limited skin penetration, with a more significant negative value indicating reduced permeation potential.

## 3 Bioavailability and metabolism of naringenin

It is becoming more widely known that gut bacteria can further increase the bioavailability of flavonoids by metabolizing them into aromatic and phenolic ring fission catabolites. This metabolic pathway of naringenin biosynthesis consists of six sequential steps, each catalyzed by specific enzymes: chalcone synthase (the primary enzyme for naringenin synthesis), phenylalanine ammonia lyase (PAL), para-coumarate-CoA ligase, cinnamate 4-hydroxylase and its associated cytochrome P450 reductase, and chalcone isomerase. Naringenin is formed by the combination of para-coumaroyl-CoA and three malonyl-CoA units. Furthermore, the initial component for naringenin production is para-coumaroyl-CoA, obtained from phenylalanine through PAL deamination in dicotyledonous plants. The second compound is hydroxylated at carbon 4 by an enzyme called cinnamate-4-hydroxylase. It is then activated by a ligase that depends on CoA. This process occurs through the phenylpropanoid pathway, a common pathway for producing flavonoids and stilbenes. In addition, monocotyledonous plants can utilize tyrosine as a substrate to directly produce p-coumaric acid without requiring the activity of the cinnamate-4-hydroxylase enzyme (Salehi et al., 2019). Absorbed naringenin travels across the lumen of the small intestine and into the colon, where it is broken down into metabolites produced by gut microbiota, including phloroglucinol and HPPA (Ucar and Goktas, 2023).

Remarkably, phloroglucinol and HPPA were also detected in gastric samples, indicating the existence of microbes in the stomach that can metabolize naringenin. Naringenin-7-O-sulfate, free naringenin, naringenin-4-O glucuronide, Aperol, and flavone-derived alcohol (flavan-4-ol) are among the main metabolites identified in the liver, the primary organ for lipid and cholesterol metabolism (Memariani et al., 2021). Interestingly, different tissues had distinct dominant metabolites. For example, the major forms of naringenin glucuronides are found in the plasma, whereas free naringenin and naringenin 7-O-sulfate are found in the GI tract, liver, and other organs. This could be due to variations in the distribution of phase-II metabolizing enzymes across tissues.

In a randomized crossover trial, participants were given orange juice as well as the same batch of fresh oranges; however, following glucuronidase and sulfatase treatment, there was an average 1.7-fold increase in naringenin when consuming fresh oranges. This indicated significant differences in naringenin’s bioavailability from different intaking oranges forms (Yang et al., 2022). Because of the fiber matrix of oranges, which makes the juice more accessible due to mechanical processing, there may be changes in the bioavailability of naringenin among various intaking forms of oranges. Additionally, because citrus flavanones are found in the inner part of the peel, they are typically avoided while consuming oranges (Pandey et al., 2015). Flavanones, such as naringin and naringenin, which are not generally ingested from whole oranges, are added to the juice through commercial juicing, which permits the albedo to come into contact with orange juice bags (Kamgaing et al., 2017). The transformation of naringenin from a promising phytocompound into a practical medicinal drug encounters numerous obstacles. Naringenin has limited absorption through the oral route owing to its low solubility in water and significant metabolism during its first passage through the liver. This restricts their optimal concentration in the bloodstream (Joshi et al., 2018). Naringenin can serve as a substrate for P-glycoprotein, a transporter that actively removes it from cells, thereby decreasing its concentration within cells and diminishing its effectiveness as a therapeutic agent. Naringenin is rapidly metabolized in the liver, forming metabolites, such as naringenin glucuronide and naringenin sulfate. These metabolites may exhibit altered or diminished biological activity compared with the original substance (Shulman et al., 2011). Maintaining therapeutic levels of naringenin is difficult because of its rapid elimination and short half-life in the bloodstream. Therefore, it is crucial to develop formulations that enhance the solubility and stability of naringenin. Novel delivery strategies such as nanoparticles, liposomes, and micelles have been investigated to improve their bioavailability (Erlund, 2002).

Another obstacle is the attainment of a regulated and prolonged release pattern for naringenin, which ensures the maintenance of optimal therapeutic concentrations throughout the duration of treatment. It is essential to develop precise delivery methods that can route naringenin to specific tissues or cancer cells while reducing any unintended effects on healthy tissues and toxicity. Extensive research and clinical studies are necessary to determine the most effective dose schedule that maximizes therapeutic efficacy while reducing adverse effects. Thorough preclinical investigations are necessary to evaluate the prolonged safety, toxicity, and adverse effects of naringenin (Mir and Tiku, 2015). Subsequent clinical trials are required to determine the safety profile of this product in human subjects. The therapeutic efficacy and safety of naringenin can be influenced by genetic variations, food, and other environmental factors, leading to changes in metabolism and reactions among various populations (Adetunji et al., 2023). To overcome these challenges, a multidisciplinary strategy is necessary to convert naringenin into a therapeutic agent. This method involved the collaboration of chemists, pharmacologists, physicians, and regulatory specialists to develop successful techniques for optimizing naringenin.

## 4 Pharmacokinetics of naringenin

The absorption of Naringenin occurs via both passive diffusion and active transport (Rani et al., 2016). Naringenin, a compound that is readily and swiftly assimilated, can be metabolized by entering the bloodstream (Arafah et al., 2020). Nevertheless, naringenin absorption through the oral cavity could be more efficient, resulting in a bioavailability of only 15%. This is because of the prolonged metabolism that occurs during the initial passage through the colon (Joshi et al., 2018; Arafah et al., 2020). Chabane et al., 2009 conducted a study on the permeability of Naringenin in human intestinal Caco-2 cells. The study found that Naringenin was partially absorbed through passive diffusion and its absorption was not influenced by pH. Furthermore, it was determined that the ATP-dependent transport substrate was promoted by multidrug resistance-associated protein (MRP1). Xu et al., 2009 conducted a study using a rat intestinal perfusion model to investigate the absorption of Naringenin. The results showed that the colon absorbed the largest amount (68%). In the same study, Naringenin exhibited an absorption rate of approximately 47% in the duodenum, 42% in the terminal ileum, and 39% in the jejunum. Kanaze et al., 2007 conducted a study to examine the pharmacokinetic parameters in humans following the administration of a 135 mg oral dosage of Naringenin. The researchers recorded a maximum concentration (Cmax) of 2009.51 ng/mL in 3.67 h after administration. The area under the concentration-time curve from 0 to infinity (AUC0−∞) was measured at 9424.52 ng h/mL. The elimination half-life was found to be 2.31 h, and its oral bioavailability was determined to be 5.81%.

Pharmacokinetic investigations of Naringenin, whether administered orally or intravenously, have indicated that its biological effects are attributed to its conjugated forms (Felgines et al., 2000; Wang et al., 2006). Naringenin mostly forms glucuronide and sulfate conjugates. Serum primarily contains naringenin glucuronides, but larger amounts of naringenin sulfates are found in tissues such as the liver, spleen, heart, and brain (Lin et al., 2014). Naringenin is present in higher amounts in tissues compared to plasma (Zeng et al., 2020). Furthermore, the presence of efflux transporters in cells does not affect naringenin (Joshi et al., 2018). The transport of Naringenin was investigated by Peng et al., 1998, who observed its uptake in the cerebral cortex and striatum, with a lower concentration in the latter. An *in vitro* model demonstrated that the apparent permeability of Naringenin ranges from 250 to 350 nm/s. Therefore, Naringenin demonstrated a significant level of permeability in both *in situ* BBB models and *in vitro* experiments. Thus, Naringenin has the potential to offer a significant level of neuroprotection to the central nervous system. The bioavailability of any substance is determined by its elimination process, which can occur through hepatic metabolism or excretion from the body, as well as by its permeability in the gastrointestinal system. The primary metabolites of flavanones identified thus far are glucuronide and sulfate conjugates (Felgines et al., 2000; El Mohsen et al., 2004; Wang et al., 2006).

After absorption, Naringenin undergoes a significant metabolic process called glucuronidation, resulting in the detection of 98% of Naringenin o β D glucuronide as a metabolite in the plasma. In addition, a significant concentration of naringenin glucuronide is present in the liver, kidney, heart, and brain. Owing to its increased polarity, naringenin glucuronide has a limited ability to penetrate tissues by crossing the lipophilic cell membrane. However, the tissue β-glucuronidase enzyme can help to break down this metabolite into free Naringenin, allowing it to be recirculated. Colonic bacterial microflora hydrolyzes Naringenin to produce 3 (4 hydroxyphenyl) propionic acid, which is readily absorbed in the gut (Day et al., 1998; Felgines et al., 2000; El Mohsen et al., 2004). Reports indicate that glucuronidation of Naringenin primarily occurs at the 7 and 4′ hydroxyl groups using the enzyme UDP-glucuronyltransferase. Therefore, sulfotransferases are involved in the O sulfation of Naringenin at the 7, 4′, or 5 hydroxyl groups (Justesen et al., 1998).

Prior to absorption in the cecum, Naringenin undergoes hydrolysis by beta-glucosidase in the small intestine (Yao et al., 2004). Naringenin undergoes further metabolism by intestinal bacterial microflora, resulting in the production of p-hydroxybenzoic acid, p-hydroxyphenylpropionic acid, and p-coumaric acid. These metabolites can be detected in both plasma and urine (Erlund et al., 2001). Flavonoids are mostly eliminated through two primary pathways: biliary and urinary excretion. The biliary excretion of metabolites (conjugates) results in their reabsorption into the enterohepatic circulation, which prolongs the half-life of the elimination phase (Ma et al., 2006). Naringenin 7-glucuronide, naringenin 7-sulfate 4′-glucuronide, and naringenin 7-glucuronide 4′-sulfate are excreted by bile, while naringenin 4′-glucuronide, naringenin 7-glucuronide, and naringenin 7,4′-disulfate are excreted through urine (Rani et al., 2016). The urine excretion rate of Naringenin varies from 7% to 23% (Joshi et al., 2018).Aging affects pharmacokinetic processes’ absorption, distribution, metabolism, and excretion phases (Sera and McPherson, 2012; Schlender et al., 2016). Aging specifically affects intestinal surface area, splanchnic blood flow, cytochrome P450 enzymatic activity, and the timing of stomach emptying and peristalsis. In terms of naringenin absorption, sex-related variations exist between rats and humans. Naringenin pharmacokinetics was also found to differ significantly based on sex in adult humans and rats (Bai et al., 2020). Notably, naringenin pharmacokinetics vary depending on the species. For instance, naringenin metabolism in the human gut was found to differ from that in rats and dogs, resulting in a notable lag time in human plasma following the oral administration of naringenin (Rebello et al., 2020). The rationale behind these variations is that they stem from variations in the body weight or body surface area of the animal species, as well as variations in metabolic processes and blood flow rate.

Variations in naringenin metabolism between species have also been reported (Isobe et al., 2018). For instance, only glucuronidation and sulfation conjugation occur in humans, whereas methylation, glucuronidation, and sulfation conjugation of metabolites are observed in rats (and dogs). Understanding the differences in naringenin and its pharmacokinetic properties among species is crucial because it facilitates the application of findings from research on non-human animals, such as rats and dogs, to human health (Burkina et al., 2016). Citrus flavanones have interesting effects on gut bacteria’s growth and gene expression and increase the number of beneficial bacteria that produce short-chain fatty acids (SCFAs). SCFAs have been demonstrated to lower inflammation, enhance the function of the intestinal barrier, and exert chemopreventive effects on colonocytes. Naringenin markedly boosted the growth and gene expression of *Bifidobacterium catenulatum* when incubated with it. These genes are involved in molecular transport, DNA repair, and cellular metabolism (de Souza et al., 2019).

## 5 Anticancer efficacy of naringenin against breast cancer

**FIGURE 1:**
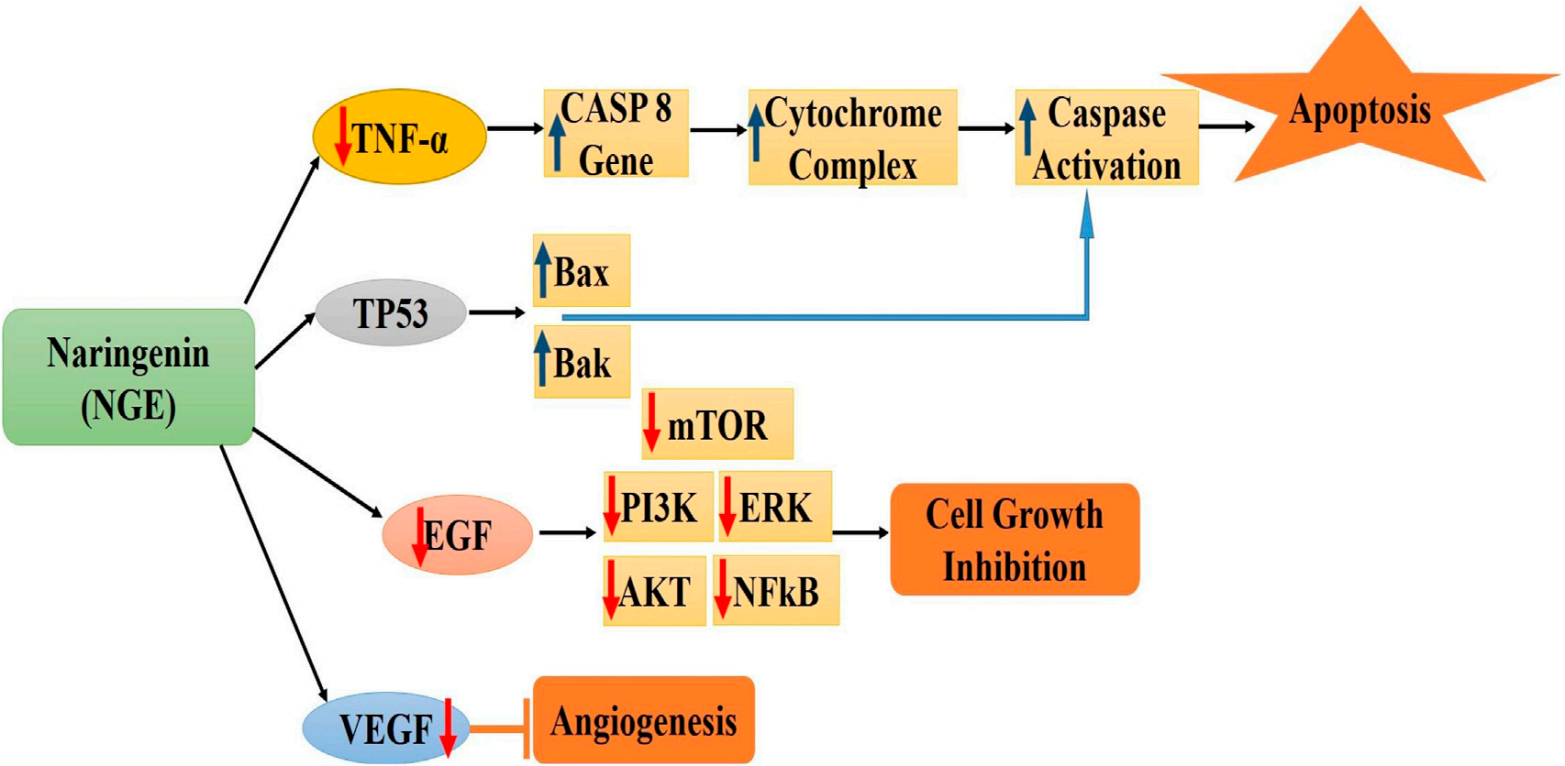
Molecular mechanisms associated with the anticancer efficacy of naringenin in breast cancer [Figures are drawn originally using MS-Powerpoint] *CASP8: Protein Coding gene; TP53: tumor protein p53; Bax and Bak: pro-apoptotic members of the Bcl-2 family; EGF: Epidermal growth factor; mTOR: mammalian target of rapamycin; ERK: Extracellular signal-regulated kinase; AKT: serine/threonine-specific protein kinase; NFkB: Nuclear factor kappa-light-chain-enhancer of activated B cells; VEGF: Vascular endothelial growth factor.

It has been demonstrated that naringenin inhibits cancer initiation, growth, and spread by altering several aberrant signaling pathways linked to inflammation, autophagy, apoptosis, proliferation, angiogenesis, invasion, and metastasis (Ponte et al., 2021). Flavonoids have anticancer properties, including apoptosis induction and cell proliferation inhibition. These effects may be due to antioxidant activity, increased carcinogen detoxification, decreased metabolic activity, inhibition of DNA adduct synthesis, and inactivation of oncogenes (Zughaibi et al., 2021). By modifying many signaling pathways, such as the Wnt/β-catenin pathway activation and EGF and TGF-β pathway blockage, naringenin prevents the proliferation and spread of cancer cells (Martínez-Rodríguez et al., 2020). Figure 1 shows the molecular processes associated with the anticancer efficacy of naringenin in breast cancer (Zhao et al., 2019).

Critical side effects, multidrug resistance, and unsuccessful medical operations are potential consequences of breast cancer therapies such as surgery, chemotherapy, radiation, and hormone therapy (van den Boogaard et al., 2022). Numerous studies have demonstrated the ability of naringenin to suppress aggressive breast tumors (Table 2). Naturally derived NGE decreases pulmonary tumor metastasis and the amount of TGF-β1 secreted by breast cancer cells (Zhang et al., 2016). It is hypothesized that naringenin possesses a high affinity for estrogen receptors (ER), namely, ER-α. Studies on triple-negative breast cancer cell lines have demonstrated that naringenin can cytotoxically reduce cell proliferation and induce cell cycle arrest in the G0/G1 phase. Apoptosis induction was evidenced by a decreased number of viable cells, growth arrest (G0/G1 phase) and nuclear condensation (Li et al., 2013; Zhao et al., 2019).

**TABLE 2:** Anticancer potential of Naringenin against Breast Cancer with mode of action.

*In vitro* (cell line)	*In vivo*	Target pathway	Mode of action	Reference
MDA-MR-231	7,12-dimethylbenz [a] anthracene (DMBA)-induced breast cancer in female rats	Mitochondrial-mediated intrinsic apoptotic pathway	Inhibited cell proliferation (time and dose-dependent)	Zhao et al. (2019)
			Arrests cell growth at G0/G1 phase	
MDA-MB-231 cells	--	Apoptotic Induction	Cell growth arrest at G2 phase of cell cycle	Wang et al. (2019)
			Inhibition of caspase (3 and 9) activity	
			Reduced viability of cancer cells	
MCF-7 cells	--	PI3K and MAPK pathways	Inhibits both PI3K and MAPK pathways	Hatkevich et al. (2014)
			Localization of ERα in cytoplasm	
			Apoptotic induction	
			Inhibited cell proliferation	
MDA-MB-231	--	JAK2/STAT3 pathway	Triggered apoptosis	Noori et al. (2020)
			Decreased cell viability	
			Upregulated expression of BAX	
			Decreased expression of Bcl-2	
			Activated Caspases (3 and 9)	
			Suppressed functioning of IL-6 in modulating apoptosis-associated genes expression	
MDA-MB-231, MDA-MB-468, BT-549 cells	MDA-MB-231 xenograft mice	β-catenin signaling pathway	Inhibited cell proliferation	Li et al. (2013)
			Promoted cell apoptosis	
			Induced G1 cycle arrest	
			Increased p21	
			Lower expression of surviving and Beta catenin	
MDA-MB-231 cells	--	Apoptotic pathway	Inhibited cell migration in dose-dependent manner	Sun and Gu (2015)
			Reduced migrant MDA-MB-231 cells	
			Decreased invasion capacity of breast cancer cells	
			Inhibited protein expressions of MMP-2 and MMP-9	
T47D-KBluc breast cancer cells	--	Eestrogenic and anti-estrogenic activities	Possessed estrogenic activity	Kim and Park (2013)
			Repressed luciferase activity	
			Increased pS2 (estrogen-regulated protein) transcription	
			Reduced TGFβ3 transcription	
MDA-MB-231 cells	--	Eestrogenic and anti-estrogenic activities	No change in pS2 mRNA expression	Kim and Park (2013)
			Slight decrease in TGFβ3 mRNA expression	
MCF10A, MCF7, T47D, BT549, MDA231, SKBR3 cells	T47D sh and T47D shNRF2 cells infected BALB C mice	FKBP4/NR3C1/NRF2 signaling pathway	Enhanced cancer cell autophagy partially owing to FKBP4/NR3C1/NRF2 axis	Xiong et al. (2022)
			Inhibited of cancer cell proliferation partially owing to the FKBP4/NR3C1/NRF2 signaling pathway	
			Induction of anti-autophagy, anti-proliferation of cancer cells	
			Pro-DC differentiation and maturation in FKBP4/NR3C1/NRF2 dependent way	
Tamoxifen Resistant MCF-7 breast cancer cells	--	PI3K and MAPK pathways	Impairs cell proliferation	Ramos et al. (2017)
			Apoptosis induction	
			Localization of ERα in perinuclear region	
			Inhibition of PI3K and MAPK signaling pathways	
MDA-MB-231 cells	--	Apoptotic pathway	Inhibited cancer cell growth	
			Induced programmed cell death	
			Induced caspase stimulation	
			Induced G2/M phase cell cycle arrest	
			Suppressed cancer metastasis	
			Reduced cell migration as well as cell invasion tendency	
	E0771 mammary tumor cells of Obese ovariectomized mice	MAPK signaling Pathway	Inhibited cancer cell growth	Ke et al. (2017)
			Increased phosphorylation of AMP-activated protein kinase (AMPK)	
			Downregulation of Cyclin D1 expression	
			Induced cell death	
			Reduced adiposity	
			Ameliorated adipose tissue inflammation	
			Moderated inhibitory effect on tumor growth in obese ovariectomized mice	

### 5.1 Antioxidant effects

Furthermore, morphological examination demonstrated that treated rats had a decreased tumor volume and a lower incidence of adenocarcinoma in the mammary gland (Mani et al., 2018). Additionally, naringenin has been shown to exhibit antioxidant effects in the tumor environment by increasing the concentrations of glutathione S-transferase, vitamin C, vitamin E, and glutathione reductase, and by suppressing the production of superoxide dismutase, catalase, thiobarbituric acid reactive substances, and nitrate (Motallebi et al., 2022). 7-O-butyl naringenin (BN), a chemically produced derivative of naringenin, has shown significant anti-proliferative chemotherapeutic potential in MCF-7 human breast cancer cells. Dose-dependent approach of BN inhibition of MCF-7 cell proliferation led to an increase in the sub-G1 phase cell population. Intracellular reactive oxygen species (ROS) were generated by BN and mitigated by pretreatment with N-acetylcysteine (NAC) (Yousuf et al., 2022). Additionally, BN increased the phosphorylation of p38, c-Jun, and stress-activated protein kinase/c-Jun NH4-terminal kinase 1/2 (SAPK/JNK1/2). However, in cells treated with BN, there was a decrease in the phosphorylation of extracellular-regulated kinase 1/2. The cytotoxicity of BN in MCF-7 cells is mediated by the activation of the p38, SAPK/JNK1/2, and c-Jun signaling pathways, in addition to the production of ROS (Park et al., 2010).

### 5.2 Apoptotic effect

Naringenin (ethanol extract of *Thymus vulgaris*) inhibits the growth of human colorectal and breast cancer cells in a manner that depends on both dose and time. This is achieved through cell cycle arrest at the S and G2/M phases, followed by an increase in apoptotic cell death. Moreover, naringenin modulates the expression of genes that control apoptosis and the cell cycle by upregulating cell cycle regulators, caspases, Bak, apoptosis-inducing factor (AIF), and Bax in breast and colorectal cancer cells. Furthermore, treatment with naringenin led to downregulation of cyclin-dependent kinases, Bcl2, X-linked inhibitor of apoptosis protein (x-IAP), and c-IAP-2. By contrast, it reduced the expression levels of NF-κB, p65, pAkt, PI3K, and pIκBα, which are factors involved in cell survival. Furthermore, NGE increases the susceptibility of breast and colorectal cancer cells to medications that act on DNA (Abaza et al., 2015).

Naringenin exhibits both antiestrogenic and estrogenic properties. Naringenin (estrogen agonist) in T47D-KBluc breast cancer cells demonstrated estrogenic efficacy in estrogen-deficient conditions and anti-estrogenic efficacy in estrogen-rich conditions. Effect of naringenin, 17-estradiol (E2), and genistein on ER activity in T47D-KBluc and ER-negative MDA-MB-231 cells was investigated. Naringenin was reported as a partial agonist and functioning as a competitive antagonist in the presence of E2 or genistein (full agonist) and inefficient ER antagonist (Kim and Park, 2013). The ability of naringenin to inhibit excessive estrogen activity and increase insufficient estrogen activity may contribute to its clinical value. Naringenin has been suggested to be a novel selective estrogen receptor modulator that may be an effective treatment for illnesses associated with sex hormones (Kim and Park, 2013).

Naringenin has demonstrated encouraging anti-metastatic characteristics in preclinical investigations (Memariani et al., 2021), suggesting its potential importance in breast cancer treatment. Naringenin affects multiple signaling pathways associated with cancer metastasis (Chen et al., 2019). For example, it hinders the PI3K/Akt and MAPK/ERK pathways, which play vital roles in cell survival, growth, and movement (Lim et al., 2017). Blocking of these pathways can result in decreased cell movement and invasiveness. In addition, naringenin regulates the NF-κB signaling system, which is linked to inflammation and metastasis, thereby enhancing its anti-metastatic properties. Naringenin has demonstrated the ability to impede the movement and infiltration of breast cancer cell lines (Sudhakaran et al., 2019). Naringenin reduces the metastatic efficacy of breast cancer cells by EMT suppression. SNS (Si-Ni-San) effectively suppressed the metastasis of breast cancer in mice exposed to chronic psychological stress (Zhang et al., 2023). A pharmacokinetic investigation demonstrated that naringenin had the most significant absorption in the hepatic tissue. Subsequent experiments conducted in living organisms and laboratory settings showed that naringenin effectively hinders the growth and spread of breast cancer caused by stress Zhang et al. (2023). This is achieved by enhancing the breakdown of estradiol through the FXR/EST signaling pathway Zhang et al. (2023).

Naringenin decreased the survival rate of MDA-MB-231 cells by causing cell cycle arrest at the G2 phase. The administration of naringenin not only affected the stage of cell cycle arrest but also triggered apoptosis in a manner that was dependent on the dosage. Treatment with naringenin also led to a substantial rise in the activity of caspase-3 and caspase-9 (*p* < 0.001). The findings of the current investigation indicate that naringenin has a suppressive impact on MDA-MB-231 cells by triggering apoptosis and restraining the activities of caspase-3 and -9 (Wang et al., 2019). Naringenin inhibits the movement of TGF-β1 from the trans-Golgi network by reducing PKC activity, leading to a decrease in the release of TGF-β1 from breast cancer cells. This discovery implies that naringenin could be a promising therapeutic option for disorders associated with TGF-β1. Naringenin demonstrated suppression of pulmonary metastasis in both 4T1/TGF-β1 tumors and 4T1/RFP cancers, leading to enhanced survival of the mice (Zhang et al., 2016).

Naringenin suppressed the rate of cell growth in a manner that was dependent on both the dosage and the duration of treatment. The AO/EB staining technique detected morphological alterations that suggest apoptotic cell death. The Annexin V/PI staining experiment demonstrated a higher proportion of apoptotic cells as the medication dosage rose. The medication naringenin was found to have the ability to induce apoptosis through the activation of caspases. Flow cytometric studies indicated cell cycle arrest, specifically at the G2/M phase of the cell cycle. The administration of the naringenin medication significantly minimized both cell migration and cell invasion propensity of MDA-MB-231 cells, as demonstrated by Qi et al. (2021).

Oral administration of naringenin dramatically reduced the number of metastatic tumor cells in the lungs and prolonged the lifespan of mice that had their tumors removed (Qin et al., 2011). Flow cytometry study demonstrated that T cells exhibited heightened antitumor efficacy in mice treated with naringenin, characterized by an elevated proportion of T cells producing IFN-γ and IL-2 (Noori et al., 2022). Additional *in vitro* investigations have shown that alleviating immunosuppression induced by regulatory T cells may be the underlying mechanism by which naringenin inhibits metastasis (Hermawan et al., 2021). The results suggest that orally administered naringenin can suppress the growth of metastases following surgery by modulating the host’s immune response (Hermawan et al., 2021). Therefore, according to Qin et al. (2011), naringenin has the potential to serve as a beneficial supplementary treatment for breast cancer patients after surgery.

## 6 Combinatorial therapy of naringenin with other therapeutic agents

In different areas of clinical practice, combination therapy, which combines many therapeutic strategies, usually yields quicker and more significant outcomes than monotherapy. Cancer is a multifactorial illness, so combination treatment aimed at many molecular targets may prove beneficial (Bhatia and Das, 2020). Recently, several pharmacological approaches to cancer treatment have been based on combination therapies (drug-food therapy and multi-drug therapy) using dietary supplements, immunotherapy, and natural products (Mokhtari et al., 2017; Guardascione and Tofoli, 2020; Sauter, 2020). Numerous treatments have targeted the estrogen receptor (ER), and more than 60% of breast tumors are ER-positive. The phosphorylation and binding of estrogens activate the ER (Prabhu et al., 2014). Although effective, anti-estrogen treatments such as tamoxifen do not specifically target the growth factor that promotes ER phosphorylation (Chang, 2012). Breast cancer cells exhibit activation of other proliferation pathways, such as the PI3K and MAPK pathways, which are linked to poor prognosis. Therefore, targeting several cellular proliferation and survival pathways at the beginning of treatment is essential to develop more effective treatments. Naringenin inhibited the MAPK and PI3K pathways (Lim et al., 2017; Cheng et al., 2020). The serum (Naringenin or Tamoxifen used charcoal-stripped serum) removed estrogen and other components. In MCF-7 breast cancer cells, combination therapy using NGE and tamoxifen was more effective than either drug alone (Hatkevich et al., 2014; Xu et al., 2018). It also significantly reduced the proliferation and viability of the cells.

Additionally, when using a combination therapy, lower concentrations of both drugs are needed to provide the same effects on viability and proliferation. In MCF-7 cells, naringenin may localize ERα to the cytoplasm and block PI3K and MAPK pathways (Ramos et al., 2017). These investigations have further helped researchers to understand the molecular processes underlying breast cancer cell division and apoptosis.

An additional investigation of combination therapy for breast cancer agents was conducted by Hatkevich et al. (2014), utilizing NGE in conjunction with the well-known medication tamoxifen. By downregulating the expression of MMP-9 and MMP-2, the combination of NGE and tamoxifen in MCF-7 cancer cells demonstrated greater efficacy with reduced cell proliferation when compared to either monotherapy. Additionally, lower quantities of both substances are needed to provide similar therapeutic effects on viability and proliferation. In addition to inducing cell death, combination treatment increases ROS generation and controls the expression of mitochondrial apoptotic proteins (Hatkevich et al., 2014). Thorough mechanistic analyses have demonstrated that tamoxifen monotherapy decreases ERα66 and GPR30 expression. However, when NGE and tamoxifen were combined, they reduced the expression of ERα66 and GPR30 and increased ERβ and ERα36, which was not the case with either monotherapy (Xu et al., 2018). The details of naringenin in breast cancer combination therapy with other agents have been summarized in Table 3.

**TABLE 3:** Studies deciphering the efficacy of naringenin with other therapeutics.

Combinatorial treatment	Cell line/*In vivo*	Mode of action	Reference
Naringenin–Tamoxifen	MCF-7 cells	Induced apoptosis	Hatkevich et al. (2014)
		Impaired pronounced cell proliferation signaling over compound alone	
Naringenin- Cyclophosphamide	MDA-MB-231 cells	Impaired proliferation signaling	Nori et al. (2020)
		Induced pronounced apoptosis over compound alone	
CUR-NGE-D-MNPs plus radiotherapy	MCF-7 cells and Tumor-bearing SD Rat	Reduced tumor volume	Askar et al. (2021)
		Cell cycle arrest	
		Apoptosis induction via modulation of CD44low, P53high, TNF-αlow, P21high, and ROS high signaling	
Naringenin + metformin + doxorubicin	MDA-MB-231 cells and 4T1 cells xenograft mouse model	Significantly increased cytotoxicity over doxorubicin alone	Pateliya et al. (2021)
		Reduced dose dependent body weight loss	
		Increased cytokines levels (TNF-α and IL-1β)	
		Improved effectiveness of doxorubicin at lower dose	
		Enhanced antitumor activity *in vivo*	
		Enhanced antitumor activity via increased necrosis and inhibited cell growth	
Naringenin + BPA (bisphenol A)	MCF-7, T47D and MDA-MB-231 cells	Impaired cancer cell proliferation	Bulzomi et al. (2012)
		Activated caspase-3 and p38	
		Prevention of BPA-induced AKT activation	
		Maintained its proapoptotic effects in the presence of BPA (food contaminant)	
Naringenin and metformin concomitant addition with doxorubicin chemotherapy	Methylnitrosourea (MNU)-induced breast cancer in rats and 4T1 cells–induced orthotopic breast cancer mouse model	Significant reduction in tumor weight and volume	Pateliya et al. (2021)
		Reduction in tumor multiplicity	
		Enhanced antitumor activity *in vivo*	
		Increased tumor necrosis	
		Combined treatment enhanced antitumor effect of doxorubicin	
U0126 (a MAPK kinase inhibitor) and Naringenin	MCF-7 cells	Impaired cell proliferation and viability	Eanes and Patel (2016)
		Greater inhibition of cell viability than either compound alone	
		Reduced protein levels of ERK1/2	
Quercetin (Que) and Naringenin (NGE)	MCF-7 cells	Induced oxidative stress	Rhman et al. (2022)
		Apoptotic induction	
		Displayed significant cytotoxicity	
		Reduced Bcl-2 gene expression	
		Increased caspase 3/7 activity	
		Increased lipid peroxidation	
		Reduced mitochondrial membrane potential (MMP)	
Quercetin/fisetin and Naringenin	MCF7 and MDA-MB-231 cells	Synergistic effect	Jalalpour et al. (2023)
		Upregulation of miR-1275 (tumor suppressor miRNA)	
		Downregulation miR-27a-3p (oncogenic miRNA)	
		Effective reduction in cancer cell growth and migration	
		Suppression and apoptosis induction	
Cryptotanshinone (CPT) + Naringenin	Mouse models of delayed type hypersensitivity (DTH)	Higher DTH	Noori et al. (2022)
		Increased lymphocyte proliferation	
		Decreased tumor growth	
		Reduced JAK2/STAT3 phosphorylation	
		Exerted immunomodulatory effects	

A new avenue for physicians to employ combination therapy to combat tumors resistant to monotherapy has been made possible by numerous studies conducted on various cancer cell lines that have shown the beneficial effects of this treatment. In a different research, Naringenin was combined with daunomycin, a therapeutically utilized medication, to overcome MCF-7 breast cancer cells’ resistance to it and increase its efficacy. Daunomycin accumulation was higher in MCF-7 (sensitive) cells than in MCF-7/ADR (resistant) cells, whereas daunomycin efflux was higher in MCF-7/ADR cells than in MCF-7 (sensitive) cells. The overexpression of P-gp in resistant cells was demonstrated by a 22-fold increase in the daunomycin IC_50_ value in MCF-7/ADR cells compared to MCF-7 (sensitive) cells. The IC_50_ values dramatically lowered when 50 μM NGE and 0.5 μM daunomycin were combined, indicating that naringenin effectively reduced daunomycin efflux in MCF-7/ADR and increased MCF-7/ADR’s resistance to daunomycin (Mir and Tiku, 2015).

## 7 Naringenin-based nanoformulations for breast cancer therapy

**FIGURE 2:**
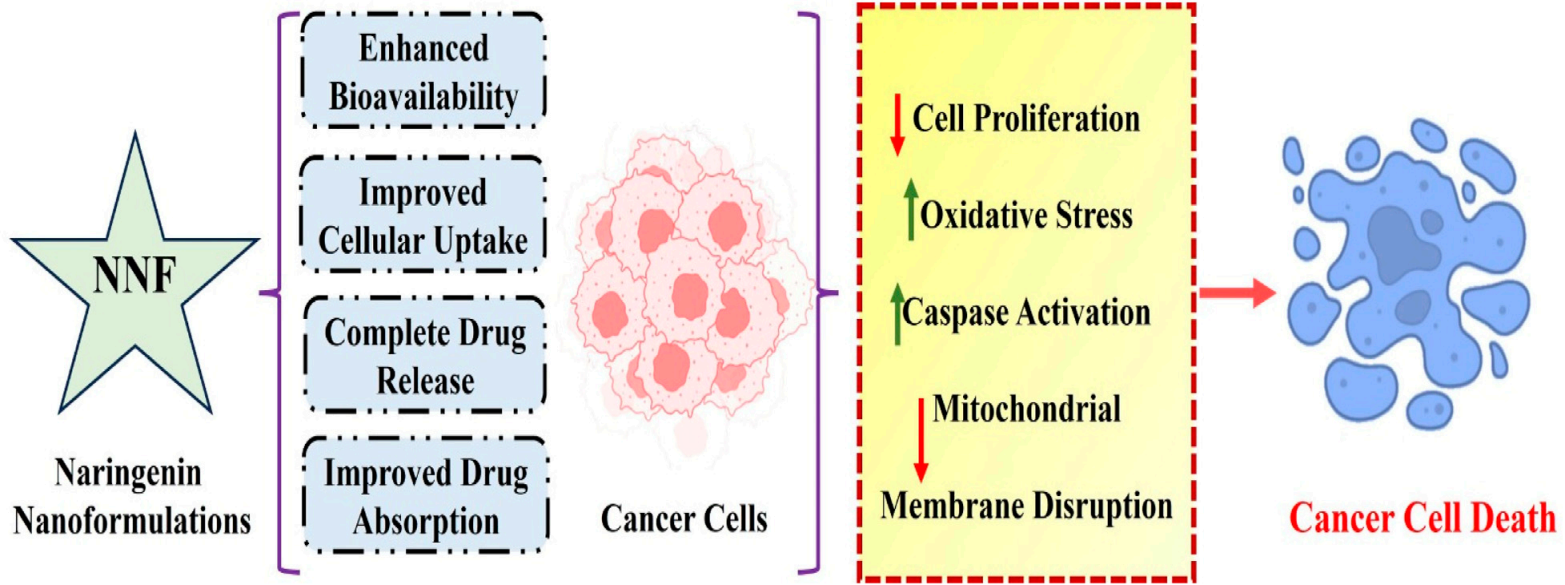
Molecular mechanisms of naringenin-based nanoformulations against breast cancer. Overall, these results highlight the enormous potential of innovative naringenin nanoformulations as effective medical delivery methods for breast cancer treatment and imply that naringeninencapsulated nanoparticles are more potent in breast cancer therapy than the solely given phytochemical agent.

Nanoparticles have several features, including thermal conductivity, a wide melting point range, and light absorption, and have been increasingly popular in the past few decades in various fields, including medical imaging and treatment tactics (Parvanian et al., 2017). Nanoscale platforms can be used as drug delivery systems to deliver medications or agents to specific tissues in a regulated manner. Additionally, as demonstrated for naringenin, nanocarriers can increase various plant-based compounds’ bioavailability and water solubility (Gera et al., 2017). Naringenin has been encapsulated in multiple nanoformulations, including micelles, hydrogels, carbon-based nanocarriers, and polymeric and lipid-based nanoparticles for treating inflammatory disorders, liver and brain diseases, and cancer therapy (Table 4 and Figure 2).

**TABLE 4:** Nanoformulations for naringenin as potent delivery systems in breast cancer therapies.

Nanoparticle	Cell line/*In vivo*	Mode of action	Reference
Curcumin-Naringenin loaded dextran-coated magnetic nanoparticles (CUR-NGE-D-MNPs)	MCF-7 cells	Induced apoptosis	Askar et al. (2021)
		Inhibited cell proliferation	
		Increased reactive oxygen species (ROS) generation	
Nanoparticles carrying Naringenin (NGESPNPs)	MCF-7 cells	Improved bioavailability	Yıldırım et al. (2022)
		Increased therapeutic efficacy	
nanosuspension of Naringenin (NGENS)	MCF-7 cells	Higher cytotoxic efficacy of NGENS	Rajamani et al. (2018)
		Increased intracellular ROS level	
		Increased mitochondrial membrane potential	
		Increased caspase-3 activity	
		Increased lipid peroxidation status (TBARS)	
		Decreased GSH (reduced glutathione levels)	
TMX-NG-SNEDDS (nano-miceller drug delivery carriers of tamoxifen with Naringenin)	MCF-7 cells and DMBA mouse model	Remarkable improvement in the rate of drug absorption and a 2-fold reduction in Tmax	Sandhu et al. (2017)
		Construed superior efficacy of the formulation via reducing tumor size	
		Improved survival rate of the animals	
		Displayed Complete drug release	

Nanoformulations of naringenin have numerous benefits compared with traditional formulations, effectively overcoming the limitations associated with its bioavailability, stability, and therapeutic effectiveness. Nanoformulations augment the surface area of naringenin particles, thus boosting their solubility in biological fluids and facilitating absorption. Nanoparticles enhance the absorption of naringenin in the gastrointestinal tract, thereby circumventing the solubility limitations of conventional formulations (Smruthi et al., 2022). Naringenin is shielded from enzymatic breakdown and acidic conditions in the stomach by encapsulating it in nanoparticles, enhancing stability and bioavailability (Chaurasia et al., 2017). Nanoformulations can be engineered to achieve a sustained release of naringenin, ensuring consistent therapeutic concentrations in the bloodstream for an extended duration and minimizing the need for frequent administration. Nanoformulations can utilize the enhanced permeability and retention (EPR) effect, whereby nanoparticles preferentially accumulate in tumor tissues due to their porous blood vessels, augmenting the concentration of naringenin at the tumor site (Paun et al., 2024). Nanoparticles can be used to overcome efflux systems, such as P-glycoprotein, which decreases the amount of naringenin inside cells, increasing its therapeutic effectiveness (Bhia et al., 2021). Nanoformulations can simultaneously encapsulate naringenin with other therapeutic compounds, enabling combination therapies that target numerous pathways simultaneously. It has the potential to enhance the efficacy of anti-cancer treatments. Nanoformulations can be designed to extend the duration of naringenin in the bloodstream, thereby improving its pharmacokinetic characteristics and ensuring long-lasting therapeutic benefits (Deshmukh et al., 2023). Nanoformulations can enhance the safety of naringenin by delivering it directly to specific tissues or cells, which reduces its overall exposure in the body and decreases the associated toxicity. This targeted delivery approach minimizes the side effects and improves the safety profile of naringenin (Sharma et al., 2022). Ultimately, nanoformulations have significant potential for addressing the drawbacks of traditional naringenin formulations by improving their solubility, stability, bioavailability, and targeted administration. Further investigation and advancement in this field will be crucial for ultimately harnessing the therapeutic capabilities of naringenin in clinical settings.

pH- and thermo-sensitive PHEMA-nanoparticles incorporating naringenin were formulated to increase cytotoxic efficacy against MCF-7 cancer cells (Yildirim et al., 2023). The maximum release of naringenin from NPs occurred at 41°C, pH 6.0. NGE-SNPs significantly decreased the viability of the cells compared to free naringenin. The NPs induced early apoptosis and increased the percentage of cells in the G1 phase of the cell cycle in a dose-dependent manner. Rajamani et al. conducted an additional experimental study to examine the effects of d-alpha tocopheryl poly (ethylene glycol) 1000 succinate (TPGS)-decorated naringenin nanoparticles on MCF-7 breast cancer cells and mouse models of cancer. These findings demonstrated the antitumor effects of naringenin nanoparticles through increased ROS levels, GSH attenuation, and caspase-3 activation, which ultimately induced apoptosis (Rajamani et al., 2018).

Naringenin has also been encapsulated using chitosan and dextran sulfate to enhance its medicinal qualities. The cytotoxic effects of spherical chitosan dextran sulfate naringenin nanoparticles on the MCF-7 breast cancer cell line were assessed using the 3-(4,5-dimethylthiazol-2-yl)-2,5-diphenyl-2H-tetrazolium bromide (MTT) assay after a 24-h incubation period. The results showed that chitosan-dextran sulfate-NGE released 80% of the free NGE in a controlled manner and exhibited significant cytotoxic action after 36 h of treatment. Therefore, the chitosan-dextran-sulfate- NGE nanocarrier is an appropriate drug delivery strategy for NGE and other hydrophobic substances (Muralidharan and Shanmugam, 2021).

The naringenin-cell penetrating peptide-galactose nanoparticles (NCG) demonstrated targeted delivery to the liver and enhanced intestinal barrier permeability in both cell and zebrafish xenotransplantation models. In addition, NCG demonstrated liver-specific targeting and enterohepatic circulation in mice breast cancer xenografts after being given orally. The cancer inhibitory effectiveness of NCG was greater than that of both NGE and the positive control tamoxifen. This was accompanied by increased hepatic EST expression and decreased estradiol levels in the liver, blood, and tumor tissue (Zhao et al., 2024).

## 8 Conclusion

Naringenin has been demonstrated to have the combined effect of inhibiting the growth of tumor cells and accelerating apoptotic cell death by activating signaling molecules and pro-apoptotic pathways in a range of distinct carcinomas. Naringenin can modulate numerous signal transduction pathways and be a suitable candidate for combinatorial therapies. There are insufficient *in vivo* studies and clinical trials, even though most cancer cell line investigations have highlighted naringenin as a viable option for treating many cancer types. Combinatorial studies of naringenin with several chemotherapeutic agents have displayed significant anticancer potential against breast cancer. Naringenin may function as a strong chemosensitizer, enhancing the cytotoxic effect of existing anticancer medications and destroying drug-resistant cancer cells. This review concludes that naringenin can reduce carcinogenesis through pleiotropic processes such as antioxidative, apoptotic-inducing ROS generation, and cell cycle arrest. However, further research is needed to elucidate the enhanced therapeutic potential of naringenin for better management of breast carcinoma.

## Abbreviations

NGE, Naringenin; HPPA, 3-(4-hydroxyphenyl) propionic acid; SCFAs, Short-chain fatty acids; BN, 7-O-butyl Naringenin; ROS, Reactive oxygen species; NAC, N-acetylcysteine; SAPK/JNK1/2, stress-activated protein kinase/c-Jun NH4-terminal kinase 1/2; MCF-7, Human breast cancer cell line with estrogen, progesterone, and glucocorticoid receptors; AIF, Apoptosis-inducing factor; x-IAP, X-linked inhibitor of apoptosis protein; c-IAP-2, Cellular inhibitor of apoptosis 2; pIκBα, Phospho-inhibitory subunit of NF Kappa B alpha; T47D-KBluc breast cancer cells, Epithelial cell line isolated from a pleural effusion of a patient with ductal carcinoma of the mammary gland; MDA-MB-231, Late-stage breast cancer cells isolated at M D Anderson from a pleural effusion of a patient with invasive ductal carcinoma; MMP-2, Matrix metalloproteinase-2; MMP-9, Matrix Metalloproteinase-9; FKBPs, cis-trans prolyl isomerase protein; NR3C1, Nuclear receptor subfamily 3 group C member 1; NRF2, Nuclear factor erythroid 2-related factor 2; BPA, Bisphenol A; MNU, Methylnitrosourea; GSH, γ-l-glutamyl-l-cysteinyl-glycine (Glutathione); TPGS, Tocopheryl polyethylene glycol succinate; MTT, 3-(4,5-dimethylthiazol-2-yl)-2,5-diphenyl-2H-tetrazolium bromide.
